# Nod2 Activates NF-kB in CD4^+^ T Cells but Its Expression Is Dispensable for T Cell-Induced Colitis

**DOI:** 10.1371/journal.pone.0082623

**Published:** 2013-12-06

**Authors:** Galliano Zanello, Ashleigh Goethel, Katharina Forster, Kaoru Geddes, Dana J. Philpott, Kenneth Croitoru

**Affiliations:** 1 Department of Medicine, University of Toronto, Toronto, Canada; 2 Department of Immunology, University of Toronto, Toronto, Canada; 3 Zane Cohen Center for Digestive Diseases, Division of Gastroenterology, Mount Sinai Hospital, Toronto, Canada; University of Chicago, United States of America

## Abstract

Although the etiology of Crohn's disease (CD) remains elusive this disease is characterized by T cell activation that leads to chronic inflammation and mucosal damage. A potential role for maladaptation between the intestinal microbiota and the mucosal immune response is suggested by the fact that mutations in the pattern recognition receptor Nod2 are associated with higher risks for developing CD. Although Nod2 deletion in CD4^+^ T cells has been shown to impair the induction of colitis in the murine T cell transfer model, the analysis of T cell intrinsic Nod2 function in T cell differentiation and T cell-mediated immunity is inconsistent between several studies. In addition, the role of T cell intrinsic Nod2 in regulatory T cell (Treg) development and function during colitis remain to be analyzed. In this study, we show that Nod2 expression is higher in activated/memory CD4^+^ T cells and its expression was inducible after T cell receptor (TCR) ligation. Nod2 stimulation with muramyl dipeptide (MDP) led to a nuclear accumulation of c-Rel NF-kB subunit. Although functionally active in CD4^+^ T cells, the deletion of Nod2 did not impair the induction and the prevention of colitis in the T cell transfer model. Moreover, Nod2 deletion did not affect the development of Foxp3^+^ Treg cells in the spleen of recipient mice and Nod2 deficient CD4 T cells expressing the OVA specific transgenic TCR were able to differentiate in Foxp3^+^ Treg cells after OVA feeding. *In vitro*, CD25^+^ Nod2 deficient T cells suppressed T cell proliferation as well as wild type counter parts and T cell stimulation with MDP did not affect the proliferation and the cytokine secretion of T cells. In conclusion, our data indicate that Nod2 is functional in murine CD4^+^ T cells but its expression is dispensable for the T cell regulation of colitis.

## Introduction

The intestinal mucosa is exposed to a large number and variety of commensal microorganisms. In pathological conditions such as inflammatory bowel diseases (IBDs), an abnormal immune response towards commensal bacteria can contribute to mucosal inflammation. Indeed, Crohn’s disease (CD) is characterized by activation of T cells that produce inflammatory cytokines and contribute to intestinal inflammation and mucosal barrier destruction. This abnormal inflammatory response is thought to result from the effect of environmental or microbial activation of the local immune response in a genetically predisposed host [1,2]. 

Mutations in the *NOD2* gene were the first defined genetic risk factors identified for CD [3,4]. Nod2 is a member of the NLR family of leucine rich repeat proteins [5-7] and is mainly expressed in dendritic cells, epithelial cells, macrophages and at a lower level in T and B cells [8-13]. Nod2 functions as a cytoplamic sensor for muramyl dipeptide (MDP), a component of bacterial peptidoglycan [14]. Upon activation with MDP, Nod2 signaling is mediated by Rip2 kinase which activates NF-kB and MAPK leading to immune gene expression [15-18]. In humans, Nod2 is functionally active in T cells and was shown to regulate Foxp3^+^ Treg cell survival by protecting from death receptor Fas-mediated apoptosis [19]. In mice, there is conflicting evidence regarding the intrinsic role of Nod2 in T cell function and in the regulation of colitis. It has been proposed that NOD2^-/-^ mice were highly sensitive to *Toxoplasma gondii* infection and that transfer of naive CD4^+^CD45RB^high^ Nod2 deficient T cells into Rag1^-/-^ recipient mice failed to induce colitis due to a T cell intrinsic defect in proliferation and Th1 differentiation [20]. However, a subsequent study showed that Nod2 deletion did not impair the development of T cell-mediated immunity against *T. gondii* and the differentiation of Th1 cells [21]. More recently, a study showed that Nod2 deletion did not affect the function of CD8^+^ T cells and the resolution of viral infection [22]. These discrepant findings led us to further investigate the intrinsic role of Nod2 in T cell function and in the induction of colitis. In addition, the role of Nod2 in Treg cell function and prevention of T cell-induced colitis remains to be analyzed. 

In this study, we show that Nod2 expression is higher in activated/memory CD4^+^ T cells and inducible after TCR ligation. Nod2 stimulation with MDP induced c-Rel nuclear translocation. Although functionally active, the deletion of Nod2 did not impair the induction and the prevention of colitis in the T cell transfer model. In addition, the *in vivo* development of Foxp3^+^ Treg cells and the *in vitro* suppressive function of CD25^+^ Treg cells were not affected by Nod2 deletion.

## Material and Methods

### Ethics statement

All mouse experiments were conducted as approved by the University of Toronto animal care committee in accordance with the regulations of the Canadian Council on animal care (University of Toronto approved protocol #20009781).

### Mice

C57BL/6 and *Rag1* ^*-/-*^ mice were purchased from The Jackson Laboratory (Bar Harbor, ME, USA), *NOD2* ^*-/-*^ mice were obtained from Dr Jean-Pierre Hugot (Hôpital Robert Debré, Université Paris Diderot, Paris, France) and *Foxp3-IRES-GFP* mice were obtained from Dr Vijay Kuchroo (Center for Neurologic Disease, Brigham and Women's Hospital, Harvard Medical School, MA, USA). Heterozygous *NOD2-GFP* mice were obtained by crossing *NOD2* ^*-/-*^ mice and C57BL/6 mice. Mice were maintained under standard pathogen-free conditions at the University of Toronto animal facility. 

### Material and reagents

The following antibodies were used for the experiments: anti-CD3ε (100331, BioLegend, San Diego, CA, USA), anti-CD3ε-FITC (11-0031-82, eBioscience, San Diego, CA, USA), anti-CD4-APC (17-0041-82, eBioscience), anti-CD4-A780 (47-0042-82, eBioscience), anti-CD8α (553027, BD Biosciences, San Jose, CA, USA), anti-CD8α-APC (17-0081-81, eBioscience), anti-TCRβ-APC-eFluor780 (47-5961-82, eBioscience), anti-CD44-PE (12-0441-82, eBioscience), anti-CD11b (553308, BD Biosciences), anti-CD25-APC (17-0251-82, eBioscience), anti-CD28 (16-0281-85, eBioscience), anti-CD45RB-PE (12-0455-82, eBioscience), anti-Foxp3-PE-Cy7 (25-5773-82, eBioscience), anti-B220 (553084, BD Biosciences), anti-Nod2 (14-5858, eBioscience), anti-c-Rel (sc-71, Santa Cruz Biotechnology, Heidelberg, Germany), anti-p65 (3034, Cell Signaling, Danvers, MA, USA), anti-β-actin (sc-130656, Santa Cruz Biotechnology), anti-RNA polymerase II (sc-899, Santa Cruz Biotechnology). MDP (tlrl-mdp) and PAM3CSK4 (tlrl-pms) were purchased from InvivoGen (InvivoGen, San Diego, CA, USA). Phorbol 12-myristate 13-acetate (PAM, P8139), ionomycin (I0634) and DNAseI were purchased from Sigma-Aldrich (Sigma-Aldrich, Saint-Louis, MO, USA). Collagenase D was purchased from Roche Applied Science (Penzberg, Germany).

### T cell transfer model of colitis and oral OVA feeding

*Rag1* ^*-/-*^ mice received an intraperitoneal (IP) injection of 3x10^5^ *NOD2* ^*-/-*^ or wild type (WT) naïve CD4^+^CD45Rb^high^ T cells with or without 1.5x10^5^ *NOD2* ^*-/-*^ or WT CD4^+^CD45Rb^low^ T cells in 500μL of phosphate-buffered saline. Mice were monitored twice weekly as described previously [23].

For oral OVA feeding experiments, *OTII-NOD2* ^*-/-*^ mice were fed OVA protein at 5mg/dose per day for 14 days by gavage on weekdays and in drinking water (1mg/ml) over the weekend.

### Cell sorting and Flow Cytometry

For T cell transfer experiments, CD4^+^ T cell subsets were isolated and sorted from spleens of C57BL/6 and *NOD2* ^*-/-*^ mice as previously described [23]. Briefly, splenocytes were stained with anti-CD4 and anti-CD45RB fluorescently labelled monoclonal antibodies and then CD4^+^CD45RB^high^ and CD4^+^CD45RB^low^ fractions were sorted on a FACSAria (BD Becton Dickinson, San Jose, CA, USA) with a purity of greater than 98%. 

Splenocytes were isolated and stained with anti-CD3, CD4 and CD25 fluorescently labelled monoclonal antibodies for 30 minutes. Cells were permeabilized with Foxp3 Fixation/Permeabilization Kit (eBioscience) and then incubated with anti-Foxp3 fluorescently labelled monoclonal antibody for 30 minutes. For FCM analysis, data were acquired on a FACSLSRII or a FACSCantoII (Becton Dickinson, Franklin Lakes, NJ, USA). Data were analyzed using FlowJo Software (Treestar, Ashland, OR, USA).

Lamina propria lymphocytes (LPLs) were isolated from the cecum of Nod2-GFP heterozygous. Cecal tissue was extracted, washed and cut into 1 to 2 cm segments that were incubated three times (37 °C, 10 minutes) in stripping buffer (PBS, 1% FBS, 5 mM EDTA, 1 mM DTT). After each incubation, the buffer was filtered through a 100-μm cell strainer and then allowed to sediment. After stripping, the tissue segments were minced and digested in digestion buffer (DMEM, 20% FBS, 2 mg/ml collagenase D, 20 μg/ml DNaseI) for two 30 minutes incubations at 37 °C. Digested material was passed through a 100-μm cell strainer, and the cells were collected by centrifugation, washed twice in DMEM and then passed through a 40-μm cell strainer to obtain LPLs. LPLs were then stained with anti-TCRβ, anti-CD4 and anti-CD44 fluorescently labelled monoclonal antibodies.

### Histological examination

Recipient *Rag1* ^*-/-*^ mice were sacrificed 12 weeks after reconstitution. The colon was opened longitudinally and separated into ascending, transverse, and descending colon and cecum. Tissues were fixed in 10% buffered formalin, sectioned, and stained with H&E. Each segment was analyzed for the severity of intestinal inflammation and graded on a scale from 0 (no change) to 4 (most severe), as described previously [24]. The scores at each segment were combined to provide an overall score of inflammation with a maximum score of 16. Weight-to-length ratios of colons were used as an objective measure of colon inflammation.

### *In vitro* T cell proliferation

CD4^+^CD25^-^ T cells were isolated from the spleen of C57BL/6 and *NOD2* ^*-/-*^ mice. Using U bottom 96 well culture plate (Sigma-Aldrich, Saint Louis, MO, USA), 2.5x10^4^ CD4^+^CD25^-^ T cells per well were cultured with 10x10^4^ *NOD2* ^*-/-*^ antigen presenting cells and 0.5µg/mL soluble anti-CD3 monoclonal antibody with or without 10µg/mL MDP for 48 hours. Proliferation was assessed by 5-bromo-2'-deoxyuridine (BrdU, Roche Diagnostics, Mannheim, Germany) incorporation as measured by luminescence. For suppression studies, CD4^+^CD25^-^ and CD4^+^CD25^+^ T cells were cultured in a 2:1 ratio for 48 hours and proliferation was assessed by BrdU incorporation. 

### Western Blotting

CD4+ T cells were isolated from the spleen of C57BL/6 and *NOD2* ^*-/-*^ mice using CD4^+^ T cell Isolation Kit (Miltenyi biotec, Bergisch Gladbach, Germany) or using a FACSAria. For assessment of Nod2 expression, 2x10^6^ CD4^+^ T cells were treated with anti-CD3 (1-10µg/mL) and anti-CD28 (2µg/mL) monoclonal antibodies for 24 hours. For assessment of c-Rel and p65 expression, 2x10^6^ CD4^+^ T cells were treated with 10µg/mL MDP for various times or 200ng/mL PMA and 300ng/mL ionomycin for 1 hour. Nuclear and cytosolic proteins were isolated using the NE-PER Nuclear and Cytoplasmic Extraction Reagents (Thermo Scientific, Waltham, MA, USA). Equal amounts of proteins were separated on SDS-PAGE and transferred onto a PVDF membrane. Membranes were incubated for 1 h at room temperature with Tris-buffered saline containing 5% bovine serum albumin (BSA) and 0.1% Tween-20 to saturate nonspecific sites. The membranes were then incubated overnight at 4°C with appropriate primary antibodies (anti-Nod2 1:250, anti-c-Rel and anti-p65 1:750, anti-β-actin 1:1000 and anti-RNA polymerase II 1:500 ) in TBS containing 0.1% Tween-20 and 5% BSA. After washing in TBS–0.1% Tween-20, the membranes were incubated for 2 hours at room temperature with a HRP-conjugated rat or rabbit anti-mouse secondary antibodies (1:1000) in TBS–0.1% Tween-20 containing 3% BSA. After washing in TBS–0.1% Tween-20, the signal was detected by SuperSignal West Pico Chemiluminescent Substrate (Thermo Scientific).

### Analysis of mRNA relative expression using quantitative real time PCR

Total RNA was isolated from T cells using RNeasy Mini Kit (Qiagen, MD, USA). RNA samples were treated with DNAse Amp I Grade (Invitrogen) and cDNA were synthesized using Mu-MLV reverse transcriptase (Eurogentec, Liège, Belgium). For RT-PCR, cDNA samples were combined with primer/probe sets and Power SYBR Green Master Mix (Applied Biosystems, Warrington, UK) according to the manufacturer's recommendations. Samples were normalized internally using simultaneously the average cycle quantification (Cq) of Ribosomal Protein L19 (RPL19) and Succinate Dehydrogenase Complex Subunit A (*SDHA*). The sequence of primers used were: *RPL19* sense GCATCCTCATGGAGCACAT, *RPL19* antisense CTGGTCAGCCAGGAGCTT, *SDHA* sense CTTGAATGAGGCTGACTGTG, *SDHA* antisense ATCACATAAGCTGGTCCTGT, *NOD2* sense CACAGGCCAACAGTCATACC, *NOD2* antisense CTCGTACTGGCTCAGAAACC. Real time assays were run on a Bio-Rad C1000 Touch Thermal Cycler (Bio-Rad, Hercules, CA, USA). Expression data are expressed as relative values after Genex macro analysis (Bio-Rad, Hercules, CA, USA). 

### Cytokine analysis

Splenic CD4^+^ T cells were cultured at 2.5x10^5^ cells per well in U bottom 96 well culture plate containing 250µL of cRPMI with plate bound anti-CD3 (10µg/mL) and soluble anti-CD28 (2µg/mL) antibodies, in presence or absence of 10µg/mL MDP and/or PAM3CSK4 for 48 hours. Cytokine secretion was measured using a Cytometric Bead Array (BD Biosciences). Data were acquired on a FACSCalibur (Becton Dickinson) and analyzed using FCAP Array software (Becton Dickinson).

### Statistical analysis

The results are depicted as mean ± SEM. Statistical analysis was performed using the unpaired Student *t* test for independent samples or 1-way analysis of variance (ANOVA) with post-test comparison. The Mann–Whitney *U* test was used for nonparametric data. Two way ANOVA was used to analyze time-course data. The differences between the 2 groups were considered significant when *P* ≤ 0.05.

## Results

### Nod2 expression is inducible in CD4^+^ T cells

**Figure 1:**
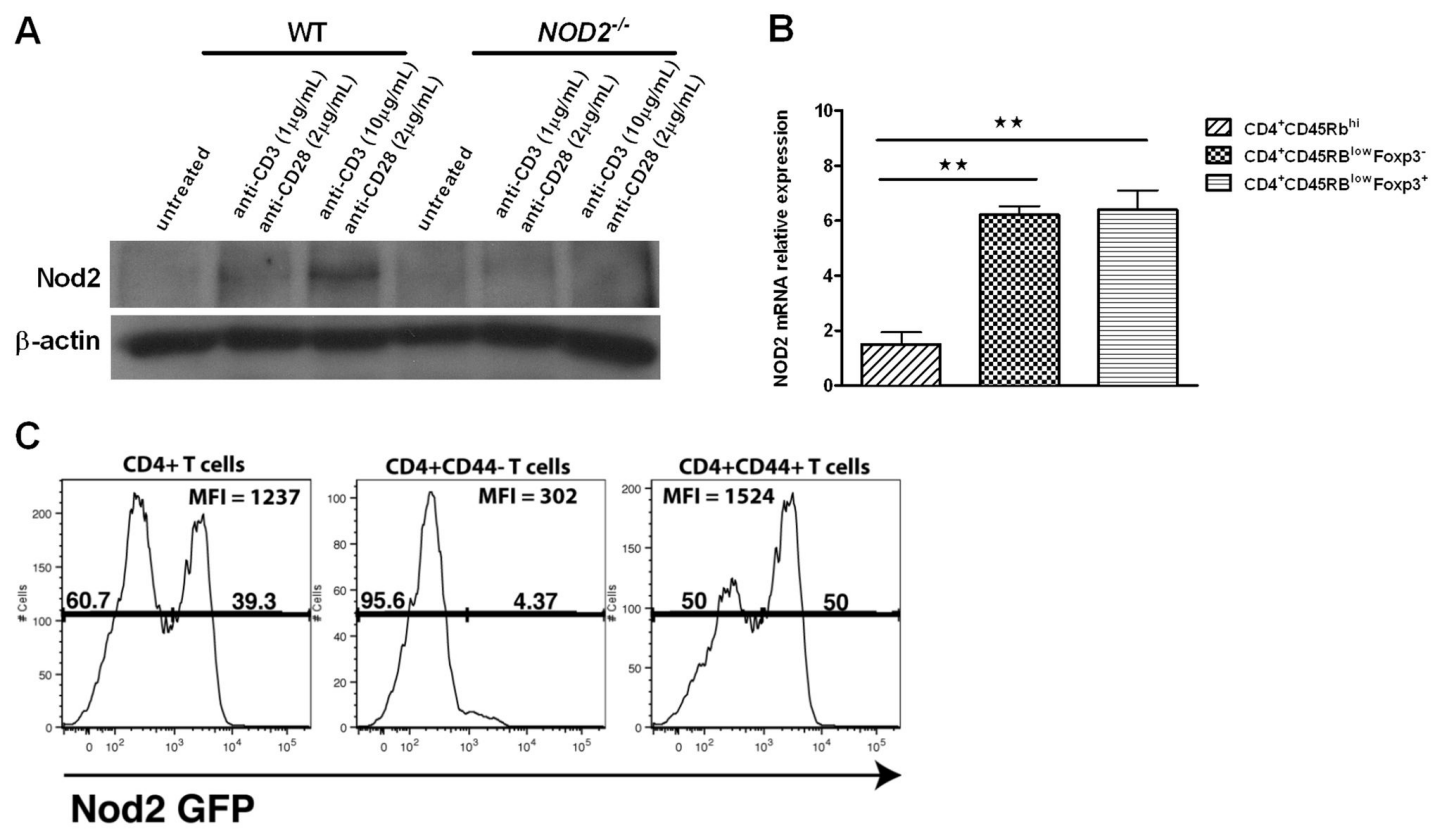
Nod2 is expressed in CD4^+^ T cells and inducible after TCR ligation. (A) Splenic CD4^+^ T cells isolated from C57BL/6 or NOD2^-/-^ mice were cultured alone or in presence of anti-CD3 (1-10µg/mL) and anti-CD28 (2µg/mL) monoclonal antibodies for 24 hours. Nod2 expression was assessed by western blot. (B) Naïve CD4^+^CD45RB^high^, effector CD4^+^CD45RB^low^Foxp3^-^ and regulatory CD4^+^CD45RB^low^Foxp3^+^ splenic T cell subsets were isolated from *Foxp3-GFP* mice and Nod2 mRNA level was assessed by RT-qPCR. (C) *Lamina propria* lymphocytes were isolated from the cecum of NOD2-GFP mice and analyzed by flow cytometry. The histograms show the level of GFP expression. Cells were gated on total viable TCRβ^+^CD4^+^ cells (left panel), on  TCRβ^+^CD4^+^CD44^-^ cells (middle panel), or TCRβ^+^CD4^+^CD44^+^ cells (right panel).

To define the role of Nod2 in the modulation of T cell function, we analyzed Nod2 expression in splenic and *lamina propria* CD4^+^ T cells. Nod2 protein expression was increased after splenic CD4^+^ T cell stimulation with anti-CD3 and anti-CD28 monoclonal antibodies, indicating that TCR ligation regulates T cell intrinsic Nod2 expression (Figure 1A). In contrast, Nod2 protein expression was not detected in CD4^+^ T cells isolated from the spleen of *NOD2* ^*-/-*^ mice (Figure 1A). Using *Foxp3-IRES-GFP* reporter mice, Nod2 mRNA expression was analyzed in different splenic CD4^+^ T cell subsets including, naive CD4^+^CD45RB^high^, activated/memory CD4^+^CD45RB^low^Foxp3^-^ and CD4^+^CD45RB^low^Foxp3^+^ T cells. Nod2 mRNA level was significantly higher in activated/memory T cells compared to naive T cells, although no differences were observed between Foxp3^-^ and Foxp3^+^ activated/memory T cell subsets (Figure 1B). This result was confirmed by measuring *GFP* expression in *lamina propria* CD4^+^ T cells isolated from the cecum of *NOD2-GFP* mice. Activated CD4^+^CD44^+^ T cells expressed a higher *GFP* level compared to CD4^+^CD44^-^ T cells (Figure 1C).

### Nod2 is functionally active in CD4^+^ T cells

**Figure 2:**
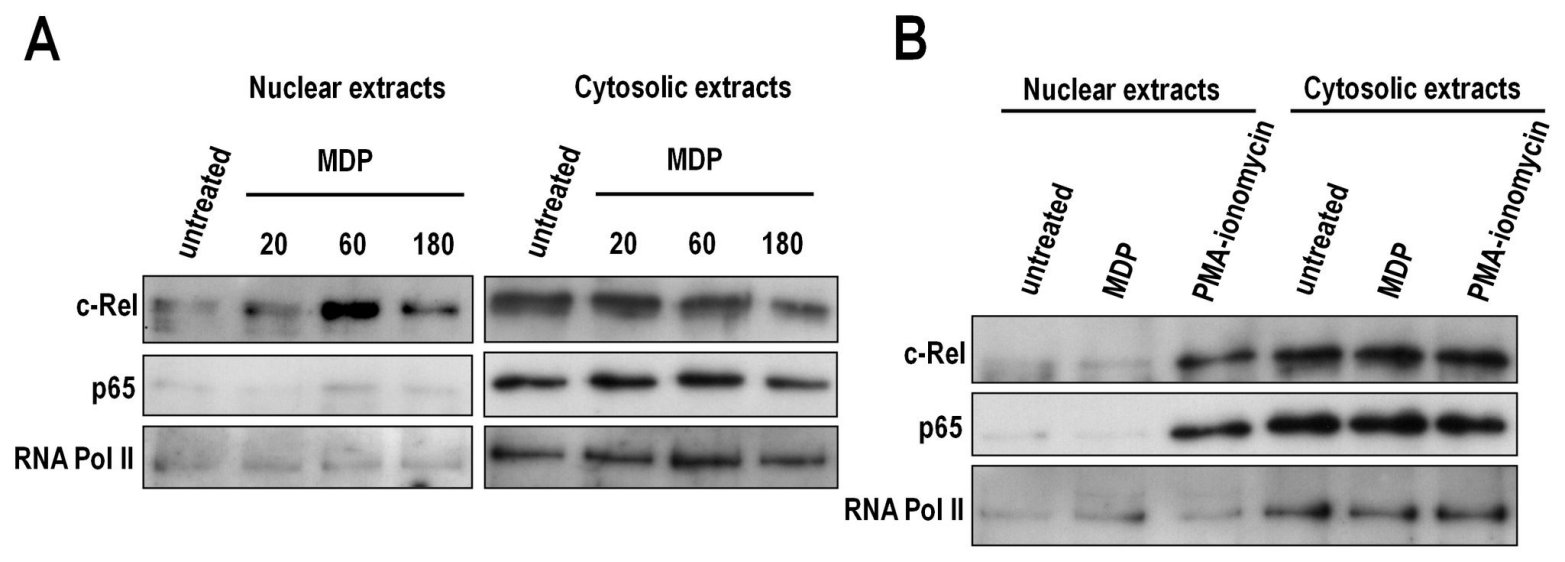
Nod2 stimulation induces c-Rel nuclear translocation in CD4^+^ T cells. Splenic CD4^+^ T cells isolated from (A) C57BL/6 or (B) *NOD2* ^*-/-*^ mice were treated with MDP (10µg/mL) for varying times or PMA (200ng/mL) and ionomycin (300ng/mL). Expression of p65 and c-Rel was assessed by western blot in both cytosolic and nuclear extracts.

We analyzed whether Nod2 stimulation could activate signaling pathways in CD4^+^ T cells. Stimulation of total splenic CD4^+^ T cells with 10μg/mL MDP was associated with the nuclear accumulation of c-Rel NF-kB subunit but not p65. The higher nuclear expression of c-Rel was observed after 60 minutes of stimulation (Figure 2A). In contrast, stimulation of Nod2 deficient CD4^+^ T cells with 10μg/mL MDP for 60 minutes was not associated with an accumulation of c-Rel in the nucleus. However, stimulation of these cells with 200ng/mL PMA and 300ng/mL ionomycin induced the nuclear accumulation of both c-Rel and p65, thus confirming that Nod2 deletion is associated with a loss of NF-kB activation after MDP stimulation (Figure 2B). 

### Nod2 deletion did not affect the induction and the prevention of colitis in the T cell transfer model

**Figure 3:**
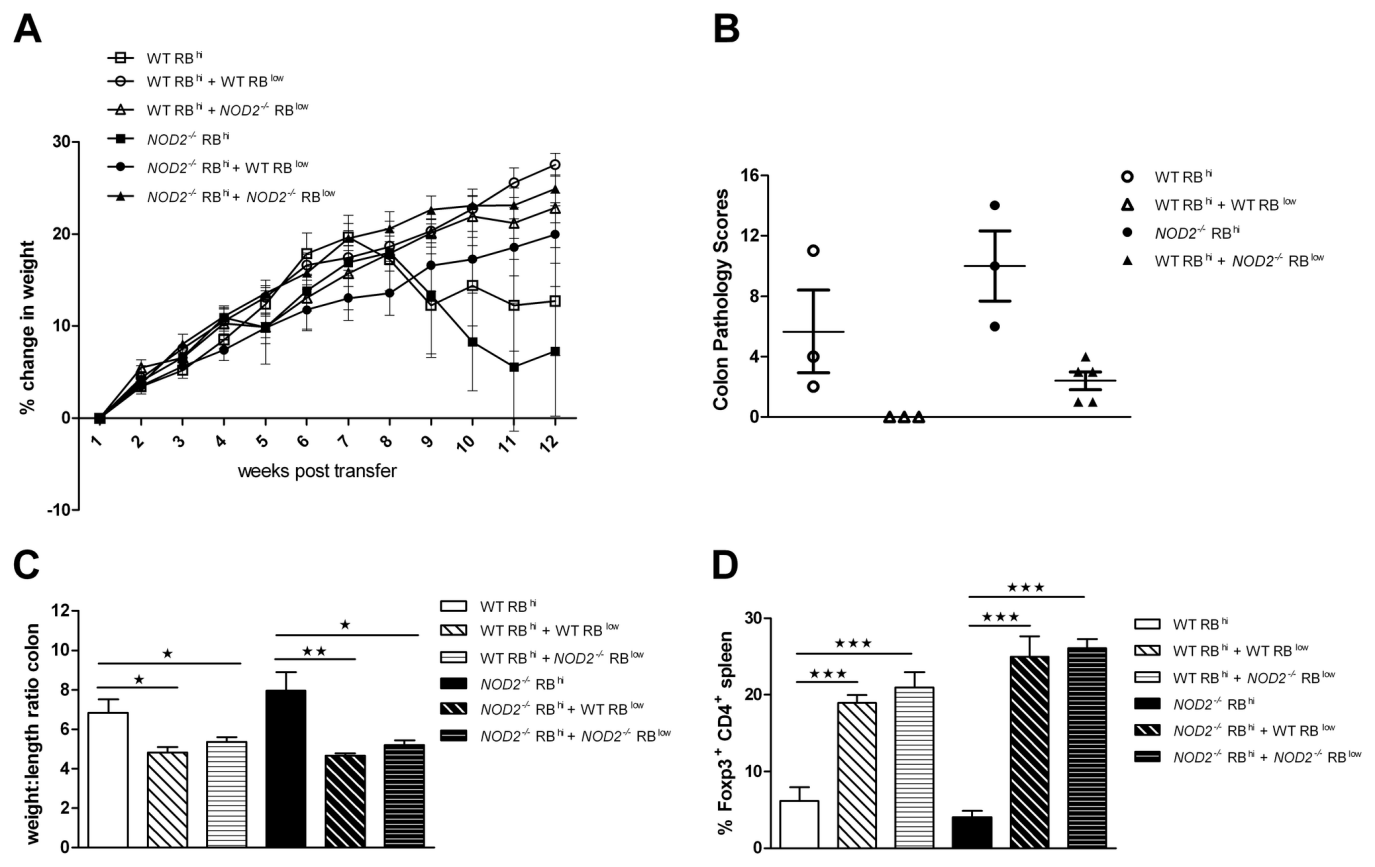
Transfer of naive and effector *NOD2* ^*-/-*^ T cells effectively induced and prevented colitis. *Rag1* ^*-/-*^ mice were transferred with 3x10^5^ wild type or *NOD2* ^*-/-*^ naïve CD4^+^CD45Rb^high^ T cells in presence or absence of CD4^+^CD45Rb^low^ T cells and (A) percentage of initial body weight change, (B) colon pathology scores and (C) colon weight to length ratio were measured. (D) Percentage of Foxp3^+^CD4^+^ T cells in the spleen of T cell transferred *Rag1* ^*-/-*^ mice. Results show the mean ± SEM and are representative of two independent experiments, n=6-9 mice per group. Colon pathology scores were assessed in one experiment, n=3-5 mice per group.

To assess whether T cell intrinsic Nod2 regulates the function of T cells in the induction or prevention of colitis, we transferred *Rag1* ^*-/-*^ recipient mice with naive CD4^+^CD45RB^high^ and activated/memory CD4^+^CD45RB^low^ T cells isolated from *NOD2* ^*-/-*^ or C57BL/6 mice. Transfer of either Nod2 deficient or wild type naive T cells induced colitis as indicated by the decreased weight gain, the higher colon pathology scores (pathology scores > 4) and the increased weight to length colon ratio observed in recipient mice (Figure 3A-C). In contrast, co-transfer of Nod2 deficient or wild type naive T cells with either Nod2 deficient or wild type activated/memory T cells suppressed the development of colitis as indicated by the increased weight gain, the lower colon pathology scores (pathology scores < 4) and the decreased weight to length colon ratio observed in recipient mice (Figure 3A-C). 

### Nod2 deletion did not alter the development or the suppressive effect of CD4^+^ T regulatory cells

**Figure 4:**
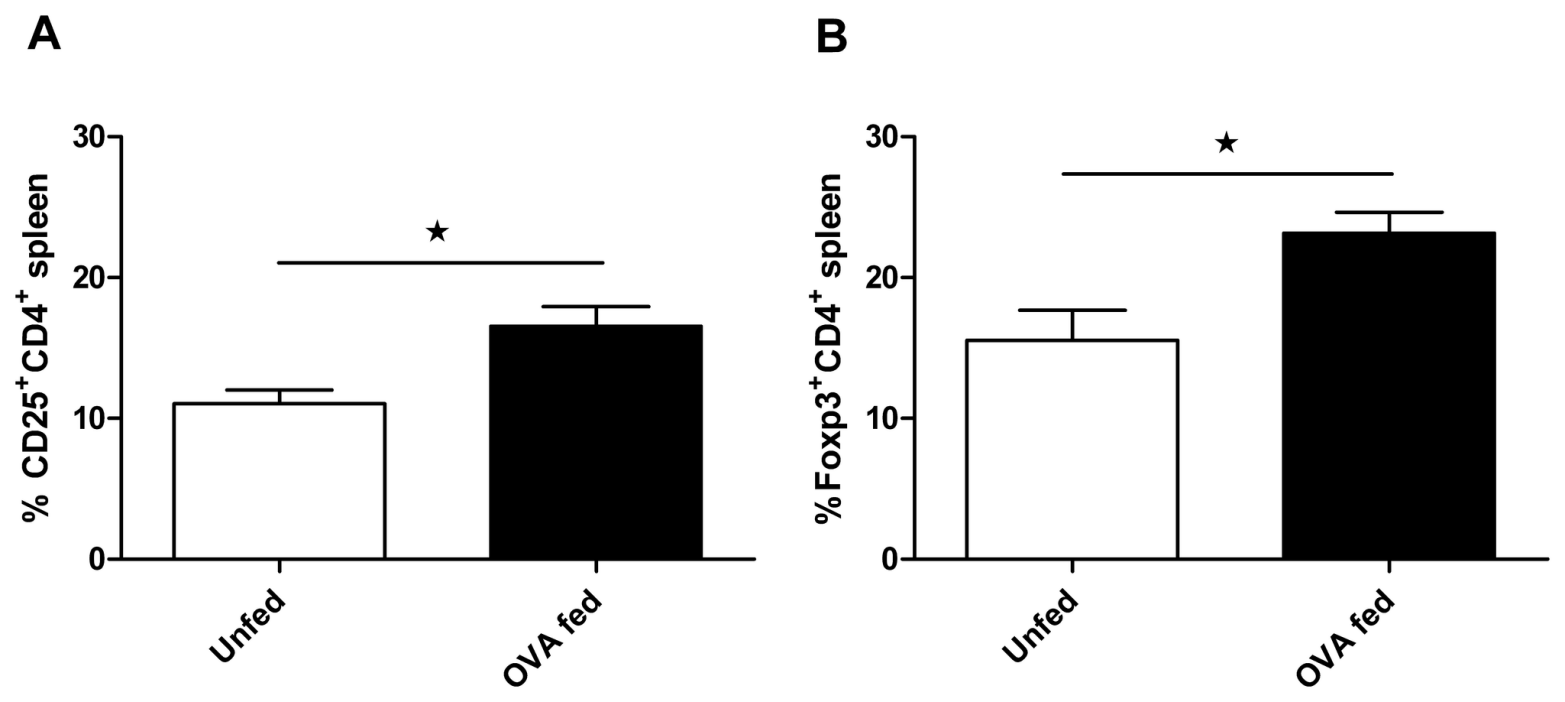
OVA feeding induced the development of CD25^+^ and Foxp3^+^ T regulatory cells in the spleen of OTII-NOD2^-/-^ mice. Mice received OVA protein at 5mg/dose per day for 14 days. Expression of CD25 and Foxp3 in splenic CD4^+^ T cells was assessed by flow cytometry.

**Figure 5:**
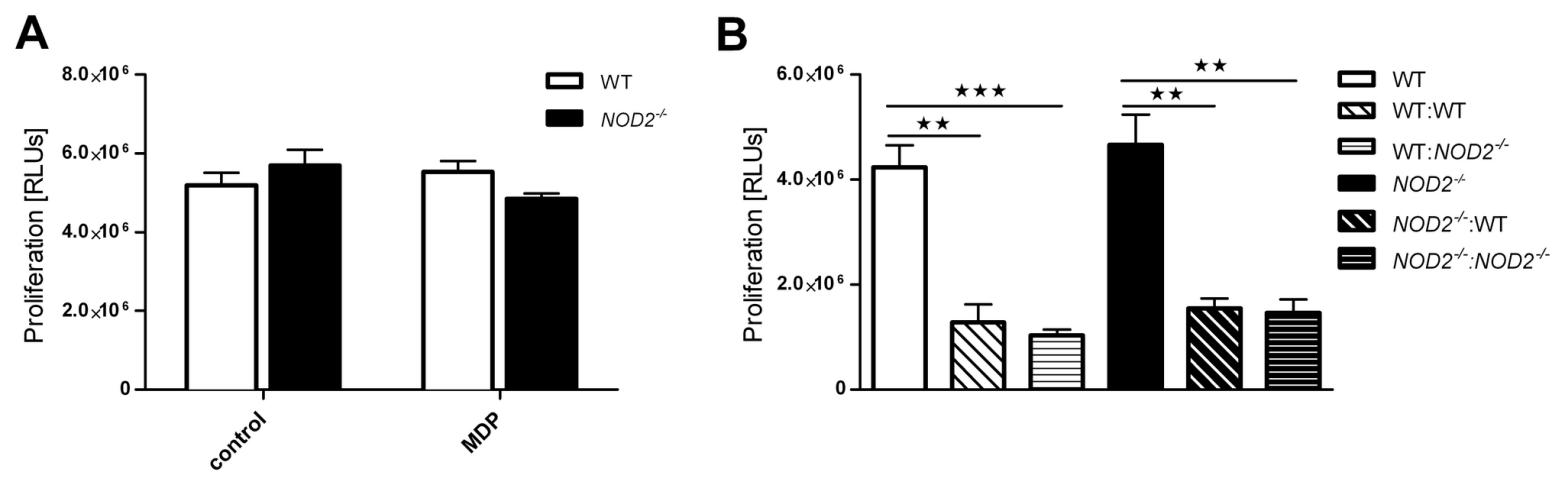
Deletion of Nod2 didn't affect the proliferation or the suppressive effect of CD4^+^ T cells. *In* *vitro* (A) proliferation and (B) suppressive effect of *NOD2* ^*-/-*^ and wild type CD4^+^ T cells. Results show the mean ± SEM and are representative of four independent experiments.

We analyzed the role of Nod2 in the development and the suppressive effect of Treg. Co-transfer of Nod2 deficient or wild type T cells was associated with a similar development of Foxp3^+^ CD4^+^ Treg cells in the spleen of *Rag1* ^*-/-*^ mice (Figure 3D). In addition, OVA feeding to *NOD2* ^*-/-*^ mice expressing an OVA transgenic TCR (*OTII/NOD2* ^*-/-*^ mice) was associated with an increase in Foxp3^+^CD4^+^ and CD25^+^CD4^+^ T cells (Figure 4). *In vitro*, proliferation of Nod2 deficient T cells was similar to wild type T cells and CD25^+^CD4^+^ Nod2 deficient T cells suppressed T cell proliferation as well as their wild type counter parts (Figure 5).

### MDP treatment did not modulate cytokine secretion in CD4^+^ T cells

**Figure 6:**
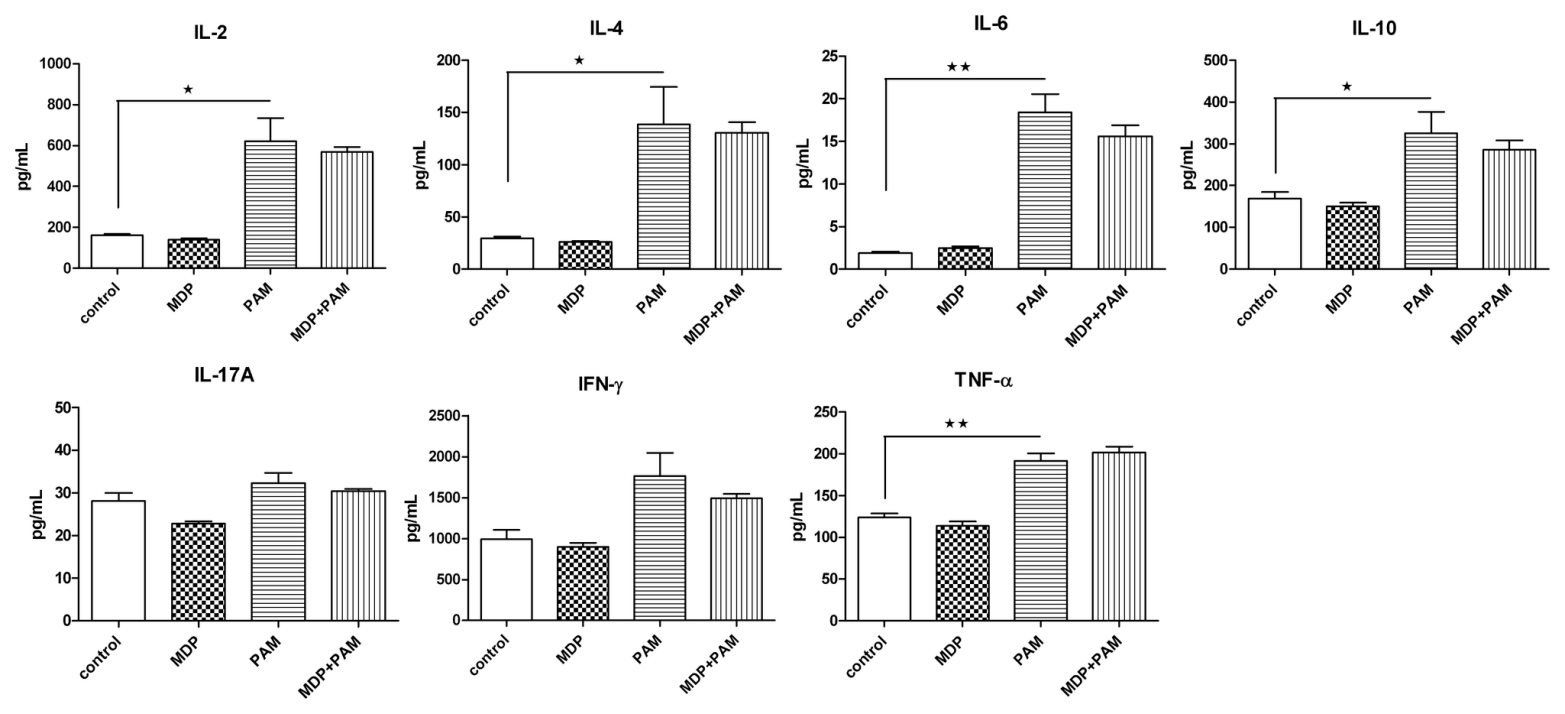
Cytokine secretion is not modulated by MDP stimulation of CD4^+^ T cells *in* *vitro*. Splenic CD4^+^ T cells isolated from C57BL/6 mice were cultured with anti-CD3 (10µg/mL) and anti-CD28 (2µg/mL) monoclonal antibodies in presence or absence of MDP (10µg/mL) and PAM3CSK4 (10µg/mL) for 48h. Cytokine secretion was quantified by cytometric bead array. Results show the mean ± SEM and are representative of three independent experiments.

Because Nod2 deletion did not affect the development of colitogenic and regulatory CD4^+^ T cells, we analyzed whether Nod2 stimulation could modulate the secretion of cytokines. Culture of total splenic CD4^+^ T cells with anti-CD3 and anti-CD28 antibodies plus 10μg/mL MDP for 48 hours did not affect cytokine secretion. In contrast, co-culture with 10μg/mL PAM3CSK4, a TLR1/2 ligand, induced a significant increase of IL-2, IL-4, IL-6, IL-10 and TNF-α. The cytokine secretion induced by PAM3CSK4 was not modulated by the addition of MDP (Figure 6). 

## Discussion

Mutations of the *NOD2* gene have been linked to higher risks for developing CD [3,4] and many studies have addressed the role of Nod2 in the regulation of innate and adaptive immunity [25]. Although it has been described that Nod2 is important for the development of T helper immunity [26-29], the analysis of the T cell intrinsic role of Nod2 has led to inconsistent results regarding its role in T cell differentiation and Th1-mediated immunity [20,21,30]. Indeed, Nod2 deletion has been shown to increase mice sensitivity to *Toxoplasma gondii* infection due to a defect in the induction of Th1-mediated immunity and transfer of naive CD4^+^CD45RB^high^ T cells deficient in Nod2 did not induce colitis in *Rag1* ^*-/-*^ mice because of a poor helper T cell differentiation and IL-2 production [20]. These results were contradicted by another report showing that Nod2 deletion did not increase sensitivity to *T. gondii* infection and did not affect Th1 and Th2 cell differentiation [21]. 

Previous reports described that Nod2 is expressed at both the mRNA and the protein level in human and murine CD4^+^ T cells [11,19,21]. Using CD4^+^ T cell subsets isolated from the spleen and the *lamina propria* of mice, we show that TCR ligation increased Nod2 expression and that activated/memory CD4^+^ T cells expressed higher level of Nod2 compared to naive CD4^+^ T cells. Moreover, Nod2 stimulation with MDP was associated with the nuclear translocation of c-Rel NF-kB subunit indicating that Nod2 is functionally active in CD4^+^ T cells. This result is reminiscent of a previous study showing that treatment of human Treg cells with MDP induced an activation of NF-kB and a nuclear translocation of the p50 and p65 subunits [19]. However, we did not observe a nuclear accumulation of p65 after stimulation of murine CD4^+^ T cells with MDP. This difference may be explained by the use of different T cell subsets and techniques used to assess NF-kB activation or due to differences between mouse and human T cells. Although activation of NF-kB plays a pivotal role in the activation, differentiation and proliferation of T cells [31,32], we observed that transfer of Nod2 deficient naive CD4^+^CD45RB^high^ T cells in *Rag1* ^*-/-*^ mice did not impair the induction of colitis. In addition, co-transfer of Nod2 deficient activated/memory CD4^+^CD45RB^low^ T cells prevented the induction of colitis and induced a development of Foxp3^+^ Treg cells comparable to the one observed after transfer of wild type T cells. *In vitro*, Nod2 deletion also did not affect T cell proliferation and CD25^+^ T cell suppressive effect. These results indicate that T cell intrinsic Nod2 is dispensable for Treg function and are in accordance with studies showing that deletion of Nod2 or Rip2, a key mediator of Nod2 signaling, did not affect T cell proliferation [21,30,33]. However, these results are inconsistent with another report describing the absence of colitis after transfer of Nod2 deficient naive T cells [20]. These contradictory results indicate that in our T cell transfer model, the deletion of Nod2 did not affect the differentiation of naive T cells into colitogenic T helper cells. The differences between our studies and those of Shaw et al. [20], might be related to differences in the microbiome composition as differences in intestinal microbiota has been shown to influence colitis severity in *NOD2* ^*-/-*^ and *NOD1* ^*-/-*^;*NOD2* ^*-/-*^ mice [34,35]. Finally, we show that although Nod2 is expressed and functionally active in CD4^+^ T cells, *in vitro* culture of T cells with anti-CD3, anti-CD28 antibodies plus MDP did not modulate cytokine secretion and did not change the PAM3CSK4-induced secretion. These results differ from a previous report showing that MDP down-regulated the cytokine secretion induced by PAM3CSK4 in splenocytes [36]. These differences may be due to fact we used an equal concentration of MDP and PAM3CSK4 while Watanabe et al. used a 20 times lower PAM3CSK4 concentration.

In conclusion, the data presented here indicate that the CD4^+^ T cell intrinsic expression of functional Nod2 is capable of modulating T cell signalling yet Nod2 is dispensable for the T cell induction and regulation of colitis and for both the development and the function of CD4^+^ Treg cells. Taken together, even though expressed and functionally active, Nod2 does not modulate the classic function of T cells thereby suggesting a more subtle function.
